# Retraction: Bone marrow mesenchymal stem cell-derived exosomal miR-25 Regulates the ubiquitination and degradation of Runx2 by SMURF1 to promote fracture healing in mice

**DOI:** 10.3389/fmed.2026.2023218

**Published:** 2026-10-30

**Authors:** Frontiers Editorial Office

The journal retracts the 7th December 2020 article cited above.

Following publication, concerns were raised regarding the integrity of the images in the published figures. The authors failed to provide the raw data or a satisfactory explanation during the investigation, which was conducted in accordance with Frontiers’ policies. Given the concerns about the validity of the data, and the lack of raw data, the editors no longer have confidence in the findings presented in the article.

This retraction was approved by the Chief Editors of Frontiers in Medicine and the Chief Executive Editor of Frontiers. The authors received a communication regarding the retraction and had a chance to respond. This communication has been recorded by the publisher.

Frontiers would like to thank the users on PubPeer for bringing the published article to our attention.

